# Supplementary material for: Recent evolutionary origin and localized diversity hotspots of mammalian coronaviruses
Source: eLife. 2024 Aug 28;13:RP91745. doi: 10.7554/eLife.91745 (PMC11357359; doi:10.7554/eLife.91745)
Supplement: Supplementary file 1. — (a) Box with definitions. Our terminology is as in Perez-Lamarque and Morlon, 2024. (b) Summary of the results obtained for the ALE reconciliations performed on “sliced mammalian phylogenetic trees”, i.e. trees where we only considered the X last Myr and merged nodes older than X Myr into polytomies in order to (i) test a scenario of more or less recent origination of coronaviruses and (ii) avoid back-in-time transfers toward nodes older than the origination time. All reconciliations are significant (when compared to randomizations shuffling host species labels). Reconciliations with an estimated number of cospeciation events larger than the estimated number of transfer events are in bold. (c) Host switches between bats and other mammal orders are less likely than expected by chance: For each type of host switches within or between orders, we reported the inferred number of host switches using ALE (on the star phylogeny) and the expected number of host switches if host switches are equally likely between species (obtained by randomly shuffling the host species names). Because we don’t have within-OTUs variations with the palmprint region, the directionality all the recent host switches is not identifiable (resulting in equal proportion in both directions at the mammalian order level). Host switches involving bats (Chiroptera) and other mammal orders are indicated in bold. (d) Frequency of host switches inferred from bats to other mammal species, including humans. For each mammal species, we computed the average number per reconciliation of host switches from bats to this mammal species. We only reported here the species presenting >10% of chance to experience at least one host switch from bats. (e) Frequency of host switches inferred from any mammal species towards humans.We computed the average number per reconciliation of host switches from each mammal species to humans. We only reported here the species presenting >10% of chance to experience at least one host sw [file elife-91745-supp1.docx]

**Supporting File 1**

Recent evolutionary origin and localized diversity hotspots of mammalian coronaviruses

Renan Maestri, Benoît Perez-Lamarque, Anna Zhukova, Hélène Morlon

Corresponding authors: Renan Maestri & Benoît Perez-Lamarque

Emails: [renanmaestri@gmail.com](mailto:xxxxx@xxxx.xxx) ; benoit.perez.lamarque@gmail.com

**This PDF file includes:**

1 supplementary box (supplementary file 1a)

7 supplementary tables (supplementary files 1b, c, d, e, f, g, and h)

**Supplementary file 1a:** Box with definitions.

Our terminology is as in Perez-Lamarque & Morlon (2024, Systematic Biology, doi: 10.1093/sysbio/syae013).

| **Codiversification/Co-cladogenesis/Co-divergence:** Pattern of concomitant diversification events happening in both host and symbiont clades. Codiversification can occur due to processes of phylogenetic tracking or successive vicariance events affecting both clades.  **Coevolution:** Process of reciprocal evolutionary changes induced by selective pressures in two (or more) interacting lineages.  **Cophylogenetic signal:** Pattern depicting the tendency of closely related species to interact with closely related partners.  **Cospeciation:** Concomitant event of host and symbiont speciations.  **Event-based methods:** Cophylogenetic methods reconciling the host and symbiont phylogenies by fitting reconciliation events (e.g. cospeciation, host transfer, duplication, or loss) on the symbiont phylogeny.  **Phylogenetic congruence:** Pattern of high similarity of the phylogenetic trees of interacting host and symbiont clades in terms of topology and relative branch lengths. If host and symbiont divergence times are matching, phylogenetic congruence can correspond to codiversification.  **Phylogenetic signal:** Pattern depicting the tendency of closely related species to have similar traits.  **Diversification by preferential host switching:** Tendency of symbionts to experience host transfers toward closely related host species; when the transfer results in a speciation event in the symbiont lineage, this tends to generate phylogenetic congruence. This does not imply co-diversification though, as the divergence times of the symbionts may be much more recent than those of the hosts. |
| --- |

**Supplementary file 1b.** **Summary of the results obtained for the ALE reconciliations performed on “sliced mammalian phylogenetic trees”, i.e.** trees where we only considered the X last Myr and merged nodes older than X Myr into polytomies in order to (i) test a scenario of more or less recent origination of coronaviruses and (ii) avoid back-in-time transfers toward nodes older than the origination time.

All reconciliations are significant (when compared to randomizations shuffling host species labels). Reconciliations with an estimated number of cospeciation events larger than the estimated number of transfer events are in bold.

| **Sliced age of the mammalian phylogeny (in Myr ago)** | **Estimated numbers of cospeciations and transfers** | **Percentage of origination in bats** | **Percentage of time-inconsistent transfers** | **Average time inconsistency (in Myr)** |
| --- | --- | --- | --- | --- |
| 55 | 119  97 | 77% | 19% | -13 Myr |
| 50 | 113  98 | 85% | 18% | -11 Myr |
| 45 | 113  100 | 84% | 17% | -11 Myr |
| 40 | 110  102 | 83% | 17% | -10 Myr |
| 35 | 102  105 | 82% | 16% | -8 Myr |
| 30 | 100  106 | 83% | 15% | -7 Myr |
| 25 | 88  109 | 40% | 14% | -6 Myr |
| 20 | 87  110 | 42% | 15% | -5 Myr |
| 15 | 75  115 | 38% | 13% | -5 Myr |
| 10 | 45  127 | 32% | 8% | -4 Myr |
| 5 | 19  153 | 40% | 1% | -1.5 Myr |

**Supplementary file 1c.** **Host switches between bats and other mammal orders are less likely than expected by chance:**

For each type of host switches within or between orders, we reported the inferred number of host switches using ALE (on the star phylogeny) and the expected number of host switches if host switches are equally likely between species (obtained by randomly shuffling the host species names). Because we don’t have within-OTUs variations with the palmprint region, the directionality all the recent host switches is not identifiable (resulting in equal proportion in both directions at the mammalian order level).

Host switches involving bats (Chiroptera) and other mammal orders are indicated in bold.

| **Type of host switch** | **Number of inferred host switches** | **Number of expected host switches if by chance** |
| --- | --- | --- |
| Chiroptera --> Chiroptera | 206 | 101 |
| Rodentia --> Rodentia | 66 | 16 |
| Artiodactyla --> Artiodactyla | 20 | 7 |
| Carnivora --> Carnivora | 14 | 4 |
| **Artiodactyla --> Chiroptera** | **11** | **28** |
| **Chiroptera --> Artiodactyla** | **11** | **28** |
| Artiodactyla --> Primates | 10 | 3 |
| Primates --> Artiodactyla | 10 | 3 |
| Primates --> Rodentia | 8 | 4 |
| Rodentia --> Primates | 8 | 4 |
| Artiodactyla --> Carnivora | 7 | 6 |
| Carnivora --> Primates | 7 | 2 |
| Primates --> Carnivora | 6 | 2 |
| Artiodactyla --> Rodentia | 5 | 11 |
| Carnivora --> Artiodactyla | 5 | 5 |
| **Chiroptera --> Rodentia** | **5** | **41** |
| **Primates --> Chiroptera** | **5** | **10** |
| Rodentia --> Artiodactyla | 5 | 11 |
| **Rodentia --> Chiroptera** | **5** | **41** |
| Artiodactyla --> Perissodactyla | 4 | infrequent |
| **Chiroptera --> Eulipotyphla** | **4** | **8** |
| **Chiroptera --> Primates** | **4** | **10** |
| **Eulipotyphla --> Chiroptera** | **4** | **7** |
| Perissodactyla --> Artiodactyla | 4 | infrequent |
| Primates --> Primates | 4 | 1 |
| Carnivora --> Rodentia | 3 | 8 |
| Rodentia --> Carnivora | 3 | 8 |
| Eulipotyphla --> Eulipotyphla | 2 | infrequent |
| Perissodactyla --> Primates | 2 | infrequent |
| Primates --> Perissodactyla | 2 | infrequent |
| Carnivora --> Perissodactyla | 1 | infrequent |
| Eulipotyphla --> Primates | 1 | 1 |
| Perissodactyla --> Carnivora | 1 | infrequent |
| Perissodactyla --> Rodentia | 1 | 1 |
| Primates --> Eulipotyphla | 1 | 1 |
| Rodentia --> Perissodactyla | 1 | 1 |
| **Carnivora --> Chiroptera** | **infrequent** | **20** |
| **Chiroptera --> Carnivora** | **infrequent** | **20** |
| Eulipotyphla --> Rodentia | infrequent | 3 |
| Rodentia --> Eulipotyphla | infrequent | 3 |
| Artiodactyla --> Eulipotyphla | infrequent | 2 |
| Eulipotyphla --> Artiodactyla | infrequent | 2 |
| **Sirenia --> Chiroptera** | **infrequent** | **2** |
| **Chiroptera --> Sirenia** | **infrequent** | **2** |
| **Chiroptera --> Pholidota** | **infrequent** | **2** |
| **Pholidota --> Chiroptera** | **infrequent** | **2** |
| **Chiroptera --> Perissodactyla** | **infrequent** | **2** |
| **Perissodactyla --> Chiroptera** | **infrequent** | **2** |
| **Lagomorpha --> Chiroptera** | **infrequent** | **2** |
| **Chiroptera --> Lagomorpha** | **infrequent** | **2** |
| Eulipotyphla --> Carnivora | infrequent | 2 |
| Carnivora --> Eulipotyphla | infrequent | 2 |
| Pholidota --> Rodentia | infrequent | 1 |
| Rodentia --> Pholidota | infrequent | 1 |
| Rodentia --> Sirenia | infrequent | 1 |
| Sirenia --> Rodentia | infrequent | 1 |
| Rodentia --> Lagomorpha | infrequent | 1 |
| Lagomorpha --> Rodentia | infrequent | 1 |
| Pholidota --> Artiodactyla | infrequent | 1 |
| Artiodactyla --> Pholidota | infrequent | 1 |
| Artiodactyla --> Sirenia | infrequent | 1 |
| Sirenia --> Artiodactyla | infrequent | 1 |
| Artiodactyla --> Lagomorpha | infrequent | 1 |
| Lagomorpha --> Artiodactyla | infrequent | 1 |

**Supplementary file 1d.** **Frequency of host switches inferred from bats to other mammal species, including humans.**

For each mammal species, we computed the average number per reconciliation of host switches from bats to this mammal species. We only reported here the species presenting >10% of chance to experience at least one host switch from bats.

| **Mammal species** | **Average number of host switches per reconciliation from bats** |
| --- | --- |
| *Homo sapiens* | 1.9 |
| *Rattus norvegicus* | 1.7288 |
| *Camelus dromedarius* | 1.4098 |
| *Sus scrofa* | 0.9996 |
| *Sorex araneus* | 0.8626 |
| *Vicugna pacos* | 0.7008 |
| *Lama glama* | 0.6912 |
| *Erinaceus amurensis* | 0.6258 |
| *Erinaceus europaeus* | 0.542 |
| *Suncus murinus* | 0.4996 |
| *Tursiops truncatus* | 0.168 |
| *Tursiops aduncus* | 0.1632 |
| *Canis lupus* | 0.1618 |
| *Mustela putorius* | 0.105 |

**Supplementary file 1e.** **Frequency of host switches inferred from any mammal species towards humans.**

We computed the average number per reconciliation of host switches from each mammal species to humans. We only reported here the species presenting >10% of chance to experience at least one host switch. Host switches from bats are highlighted in bold.

| **Mammal species** | **Average number of host switches per reconciliation towards humans** |
| --- | --- |
| *Camelus dromedarius* | 0.41 |
| *Mus musculus* | 0.31 |
| *Canis lupus* | 0.26 |
| *Sus scrofa* | 0.24 |
| *Suncus murinus* | 0.22 |
| *Paradoxurus hermaphroditus* | 0.21 |
| *Paguma larvata* | 0.21 |
| *Chlorocebus aethiops* | 0.21 |
| *Vicugna pacos* | 0.20 |
| ***Hipposideros vittatus*** | **0.17** |
| *Pan troglodytes* | 0.17 |
| *Bos taurus* | 0.17 |
| ***Rousettus aegyptiacus*** | **0.16** |
| ***Hipposideros abae*** | **0.16** |
| ***Hipposideros ruber*** | **0.15** |
| *Rattus losea* | 0.14 |
| *Rattus norvegicus* | 0.14 |
| *Myodes rufocanus* | 0.13 |
| *Equus caballus* | 0.13 |
| *Rattus tanezumi* | 0.12 |
| *Odocoileus virginianus* | 0.12 |
| *Rattus argentiventer* | 0.11 |
| *Neovison vison* | 0.10 |
| *Bos grunniens* | 0.10 |
| *Mustela putorius* | 0.10 |

**Supplementary file 1f.** Summary of the different strategies used to evaluate the robustness of our findings.

| **Potential bias or issues** | **Solution** | **Analyses** |
| --- | --- | --- |
| Over-sampling of humans and domesticated animals | Subsampling of the dataset to only 3 Genbank accessions per mammal species | PhyloBayes + ALE on each of the 50 subsampled datasets |
| Over-representation of bats in the dataset | Subsampling of the dataset (up to 10 species per mammalian order only) | PhyloBayes + ALE on each of the 50 subsampled datasets |
| The palmprint region of the RdRp region may be subject to recombination | Split of the palmprint region into two 150-amino acid subparts | PhyloBayes + ALE on each subpart |
| Coronavirus origination is spuriously inferred at the origin of Pteropodidae | Permutations of the host species in the mammal phylogeny (1) randomly or (2) by constraining by bioregion | PhyloBayes + ALE on each of the 100 randomized datasets |
| Origination cannot be inferred using a star phylogeny for the reconciliation | Simulations of scenarios of diversification per preferential host switches | PhyloBayes + ALE on each of the 50 simulated datasets |
| Observing 20% of time-inconsistent host switches can happen under a scenario of codiversification | Simulations of scenarios of codiversification | PhyloBayes + ALE on each of the 50 simulated datasets |
| Higher diversification rates of coronaviruses within bats can generate a spurious inference of an origination in bats | Simulations of scenarios of origination in rodents and diversification per preferential host switches with higher diversification rates of coronaviruses within bats | PhyloBayes + ALE on each of the 50 simulated datasets |

**Supplementary file 1g.** **Results are qualitatively similar when running ALE on sub-parts on the palmprint region.**

We reported here results obtained when running ALE on the star phylogeny on (i) the whole palmprint region (positions 1-150), (ii) the first part of the palmprint region (positions 1-75) or (iii) the last part of the palmprint region (positions 76-150).

|  | **Whole palmprint region**  **(positions 1-150)** | **First part of the palmprint region (positions 1-75)** | **Last part of the palmprint region (positions 76-150)** |
| --- | --- | --- | --- |
| Percentage of time-inconsistent host switches | 21% | 24% | 22% |
| Percentage of originations in bats | 56% | 46% | 64% |
| Percentage of within-order host switches | 68% | 70% | 68% |
| Percentage of host switches from bats to others (and *vice versa*) | 10% | 12% | 11% |

**Supplementary file 1h.** Mammal silhouettes taken from open-to-use sources in phylopic.org, detailed credits for authors given below.

| **Mammal** | **Author** |
| --- | --- |
| Asian palm civet | Margot Michaud |
| Bat | Yan Wong |
| *Camelus dromedarius* | Steven Traver |
| *Equus caballus* | Jody Taylor |
| *Homo sapiens* | 1. Michael Keesey |
| *Mus musculus* | Kamil S. Jaron |
| *Oryctolagus cuniculus* | Steven Traver |
| Pangolin | Steven Traver |
| *Sorex araneus* | Becky Barnes |
| Trichetus | Steven Traver |
